# Illustrations of Homœopathy

**Published:** 1856-04

**Authors:** 


*Illustrations of Homœopathy.*—It is not often that we take up our space with this subject. Homœopathy speaks for itself, and we presume that most of our readers could bring forward "illustrations" similar to the following, which have occurred under their own observation.

The case of the late Emperor of Russia is also in point. It will be remembered that he was attended by a homœopathist, and that there has been much uncertainty as to the true nature of his disease, and speculation on the cause of his sudden death. The following may, perhaps, be the true interpretation of the case. "Mr. Makley, surgeon and coroner of London, states that the official document, reporting the death of the Emperor Nicholas, describes *an impossibility*. That document states that the Emperor died of paralysis of the lungs and bronchitis; and that at the last he took leave of his family *with a firm voice*, which Mr. Makley says was *physically impossible*, if he was suffering from the disease stated in the certificate of death.

"The belief, therefore, on the minds of the medical men in this country, was, that the Emperor was poisoned; and it is my own opinion, not that he was poisoned by those about him, but that he committed suicide."—*N. Y. Med. Reporter.*

*Homœopathic Trickeries.*—Dr. Coale relates the following facts. He was sent for to visit a child with convulsions, one afternoon lately. Being absent at the time, he did not see the child until the next morning. He then found that a homœopathic practitioner had been in attendance, and had given *wine of ipecac*, in the dose of *two teaspoonfuls*, at the same time leaving directions that if the child did *not* have any more convulsions, it should be made to swallow two or three of the little *pellets* left by him; if it *had* any more attacks, *it must have the wine of ipecac again!*

Dr. C. said that these facts, in themselves, were unimportant, save in so far as they illustrate the deception of this class of practitioners, who, while they hold out the idea to their employers that they are giving infinitely small doses, and that this is the only safe practice, often use the preparations and doses given by regular physicians. Similar occurrences are getting to be so frequent that they should be exposed, in simple justice to the profession.

Dr. C. also mentioned that whilst varying the doses of the biniodide of mercury very gradually to one-eighth of a grain, in a certain case, the apothecary who put up the prescription, showed him another, of a homœopathic practitioner, in which the dose was one-sixth of a grain of biniodide of mercury combined with three grains of hydriodate of potassa, four times a day. Truly *infinitesimal.—Boston Med. and Surg. Journ.*

